# Supplementary material for: NeuroBooster Array: A Genome-Wide Genotyping Platform to Study Neurological Disorders Across Diverse Populations
Source: medRxiv. 2023 Nov 14:2023.11.06.23298176. Preprint. [Version 2] doi: 10.1101/2023.11.06.23298176 (PMC10659467; doi:10.1101/2023.11.06.23298176)
Supplement: Supplement 3 [file media-3.pdf]

Global Parkinson's Genetics Program (GP2) Banner Authors

This project was supported by the Global Parkinson's Genetics Program (GP2). GP2 is funded by the Aligning Science Across Parkinson's (ASAP) initiative and implemented by The Michael J. Fox Foundation for Parkinson's Research. For a complete list of GP2 members see https://gp2.org.

Members: 235
Version date: 18 August 2023

Argentina	Emilia M Gatto	Sanatorio de la Trinidad Mitre- INEBA	Nothing to declare
	Marcelo Kauffman	Hospital JM Ramos Mejia	Nothing to declare
Armenia	Samson Khachatryan	Somnus Neurology Clinic	Nothing to declare
	Zaruhi Tavadyan	Somnus Neurology Clinic	Nothing to declare
Australia	Claire E Shepherd	Neuroscience Research Australia	The Sydney Brain Bank is located at and supported by Neuroscience Research Australia
	Julie Hunter	ANZAC Research Institute	Nothing to declare
	Kishore Kumar	Garvan Institute of Medical Research and Concord Repatriation General Hospital	Paul Ainsworth Family Foundation
	Melina Ellis	Concord Hospital	Nothing to declare
	Miguel E. Rentería	QIMR Berghofer Medical Research Institute	The Australian Parkinson's Genetics Study is supported by the Shake It Up Australia Foundation and The Michael J. Fox Foundation for Parkinson's Research
	Sulev Koks	Murdoch University	Nothing to declare
Austria	Alexander Zimprich	Medical University Vienna Austria	Nothing to declare
Brazil	Artur F. Schumacher-Schuh	Universidade Federal do Rio Grande do Sul / Hospital de Clínicas de Porto Alegre	Nothing to declare
	Carlos Rieder	Federal University of Health Sciences of Porto Alegre	Nothing to declare
	Paula Saffie Awad	Universidade Federal do Rio Grande do Sul	Global Parkinson's Genetics Program
	Vitor Tumas	University of São Paulo	Nothing to declare
	Sarah Camargos	Universidade Federal de Minas Gerais	Nothing to declare
Canada	Edward A. Fon	Montreal Neurological Institute	Nothing to declare

	Oury Monchi	Institut universitaire de gériatrie de Montréal	CIHR, Brain Canada, Parkinson Canada
	Ted Fon	McGill University	Nothing to declare
Chile	Benjamin Pizarro Galleguillos	Universidad de Chile	Nothing to declare
	Marcelo Miranda	Fundación Diagnosis	Nothing to declare
	Maria Leonor Bustamante	Faculty of Medicine Universidad de Chile	Nothing to declare
	Patricio Olguin	Universidad de Chile	Nothing to declare
	Pedro Chana	CETRAM	Nothing to declare
China	Beisha Tang	Central South University	Nothing to declare
	Huifang Shang	West China Hospital Sichuan University	Nothing to declare
	Jifeng Guo	Xiangya Hospital	Nothing to declare
	Piu Chan	Capital Medical University	Nothing to declare
	Wei Luo	Zhejiang University	Nothing to declare
Colombia	Gonzalo Arboleda	Universidad Nacional de Colombia	Nothing to declare
	Jorge Orozco	Fundación Valle del Lili	Nothing to declare
	Marlene Jimenez del Rio	University of Antioquia	Nothing to declare
Costa Rica	Alvaro Hernandez	University of Costa Rica	Nothing to declare
Egypt	Mohamed Salama	The American University in Cairo	The AUC/ ASRT/ DAAD
	Walaa A. Kamel	Beni-Suef University	Nothing to declare
Ethiopia	Yared Z. Zewde	Addis Ababa University	Nothing to declare
France	Alexis Brice	Paris Brain Institute	Nothing to declare
	Jean-Christophe Corvol	Sorbonne Université	Nothing to declare
Germany	Ana Westenberger	University of Lübeck	Nothing to declare
	Anastasia Illarionova	Deutsches Zentrum für Neurodegenerative Erkrankungen	Nothing to declare
	Brit Mollenhauer	University Medical Center Göttingen	Nothing to declare
	Christine Klein	University of Lübeck	CK serves as a medical Advisor to Centogene on genetic testing reports in the field of movement disorders, except Parkinson's disease, and is a member of the Scientific Advisory Board of Retromer Therapeutics
	Eva-Juliane Vollstedt	University of Lübeck	Nothing to declare
	Franziska Hopfner	Department of Neurology, University Hospital, LMU Munich	Nothing to declare
	Günter Höglinger	Department of Neurology, University	Nothing to declare

		Hospital, LMU Munich	
	Harutyun Madoev	University of Lübeck	Nothing to declare
	Joanne Trinh	University of Lübeck	Nothing to declare
	Johanna Junker	University of Lübeck	Johanna Junker is funded by the MJFF Data Community Innovators Program and received a Family Mobility Grant from the University of Luebeck
	Katja Lohmann	University of Lübeck	Nothing to declare
	Lara M. Lange	University of Lübeck and University Medical Center Schleswig-Holstein	Nothing to declare
	Manu Sharma	University of Tubingen	Dr. Sharma is further funded by the Michael J Fox Foundation, USA Genetic Diversity in PD Program: GAP-India Grant ID: 009411.
	Sergiu Groppa	University of Mainz	Nothing to declare
	Thomas Gasser	University of Tübingen	Nothing to declare
	Zih-Hua Fang	The German Center for Neurodegenerative Diseases	Nothing to declare
Ghana	Albert Akpalu	University of Ghana Medical School	Nothing to declare
Greece	Georgia Xiromerisiou	University of Thessaly	Nothing to declare
	Georgios Hadjigorgiou	University of Thessaly	Nothing to declare
	Ioannis Dagklis	Aristotle University of Thessaloniki	Nothing to declare
	Ioannis Tarnanas	Ionian University	Nothing to declare
	Leonidas Stefanis	Biomedical research Foundation of the Academy of Athens	PPMI2 (funded by MJFF), ALAMEDA (H2020 grant), funding by HFRI
	Maria Stamelou	Diagnostic and Therapeutic Centre HYGEIA Hospital	The non-profit organisation for scientific research in Parkinson’s disease and related disorders
	Efthymios Dadiotis	University of Thessaly	Nothing to declare
Honduras	Alex Medina	Hospital San Felipe	Nothing to declare
Hong Kong	Germaine Hiu-Fai Chan	Queen Elizabeth Hospital	Nothing to declare
	Nancy Ip	The Hong Kong University of Science and Technology	Nothing to declare
	Nelson Yuk-Fai Cheung	Queen Elizabeth Hospital	Nothing to declare
	Phillip Chan	The Hong Kong University of Science and Technology	Nothing to declare
	Xiaopu Zhou	The Hong Kong University of Science and Technology	Nothing to declare
India	Asha Kishore	Aster Medcity	Michael J. Fox Foundation
	Divya KP	Sree Chitra Tirunal	Nothing to declare

		Institute for Medical Sciences and Technology	
	Pramod Pal	National Institute of Mental Health & Neurosciences	Nothing to declare
	Prashanth Lingappa Kukkle	Manipal Hospital	Nothing to declare
	Roopa Rajan	All India Institute of Medical Sciences	Nothing to declare
	Rupam Borgohain	Nizam's Institute Of Medical Sciences	Nothing to declare
Iran	Mehri Salari	Shahid Beheshti University of Medical Science	Nothing to declare
Italy	Andrea Quattrone	Magna Græcia University of Catanzaro	Nothing to declare
	Enza Maria Valente	University of Pavia	Nothing to declare
	Lucilla Parnetti	University of Perugia	Nothing to declare
	Micol Avenali	University of Pavia	Nothing to declare
	Tommaso Schirinzi	University of Rome Tor Vergata	Nothing to declare
Japan	Manabu Funayama	Juntendo University	Nothing to declare
	Nobutaka Hattori	Juntendo University faculty of medicine	Nothing to declare
	Tomotaka Shiraishi	Jikei University School of Medicine	Nothing to declare
Kazakhstan	Altynay Karimova	Institute of Neurology and Neurorehabilitation	Nothing to declare
	Gulnaz Kaishibayeva	Institute of Neurology and Neurorehabilitation	Nothing to declare
Kyrgyzstan	Cholpon Shambetova	Kyrgyz State Medical Academy	Nothing to declare
Luxembourg	Rejko Krüger	University of Luxembourg	Nothing to declare
Malaysia	Ai Huey Tan	University of Malaya	Nothing to declare
	Azlina Ahmad-Annuar	University of Malaya	Nothing to declare
	Mohamed Ibrahim Norlinah	Universiti Kebangsaan Malaysia	Nothing to declare
	Nor Azian Abdul Murad	UKM Medical Molecular Biology Institute	Nothing to declare
	Shahrul Azmin	Universiti Kebangsaan Malaysia Medical Centre	Nothing to declare
	Shen-Yang Lim	University of Malaya	Nothing to declare
	Wael Mohamed	International Islamic University	Nothing to declare
	Yi Wen Tay	University of Malaya	Nothing to declare
Mexico	Daniel Martinez-Ramirez	Tecnologico de Monterrey	Nothing to declare

	Mayela Rodriguez-Violante	Instituto Nacional de Neurologia y Neurocirugia	Nothing to declare
	Paula Reyes-Pérez	Universidad Nacional Autónoma de México	Global Parkinson's Genetics Program
Mongolia	Bayasgalan Tserensodnom	Mongolian National University of Medical Sciences	Nothing to declare
Nepal	Rajeev Ojha	Tribhuvan University	Nothing to declare
New Zealand	Tim J. Anderson	University of Otago	Heath Research Council of New Zealand; Ministry of Business Innovation and Employment (New Zealand), Neurological Foundation of New Zealand
	Toni L. Pitcher	University of Otago	Funding - Health Research Council of New Zealand
Nigeria	Arinola Sanyaolu	University of Lagos	Nothing to declare
	Njideka Okubadejo	University of Lagos	Michael J Fox Foundation; Tertiary Education Trust Fund (TETFUND) National Research Fund
	Oluwadamilola Ojo	College of Medicine of the University of Lagos	Nothing to declare
Norway	Jan O. Aasly	Norwegian University of Science and Technology	Nothing to declare
	Lasse Pihlstrøm	Oslo University Hospital	Southeastern Regional Health Authority, Norway and Michael J. Fox Foundation
	Manuela Tan	Oslo University Hospital	Southeastern Norway Regional Health Authority Norway and Michael J. Fox Foundation
Pakistan	Shoaib Ur-Rehman	University of Science and Technology Bannu	Nothing to declare
Peru	Mario Cornejo-Olivas	Universidad Cientifica del Sur	Michael J. Fox Foundation for Parkinson's Research and Aligning Science Across Parkinson's Initiative
Philippines	Maria Leila Doquenia	Metropolitan Medical Center	Nothing to declare
	Raymond Rosales	Metropolitan Medical Center	Nothing to declare
Puerto Rico	Angel Vinuela	University of Puerto Rico	Nothing to declare
Russia	Elena Iakovenko	Research Center of Neurology	Nothing to declare
Saudi Arabia	Bashayer Al Mubarak	King Faisal Specialist Hospital and Research Center	Nothing to declare
	Muhammad Umair	King Abdullah International Medical Research Center	Nothing to declare
Singapore	Eng-King Tan	National Neuroscience Institute	Singapore National Medical Research Council (MOH-OFLCG-000207)
	Jia Nee Foo	Nanyang Technological University	Singapore National Medical Research Council (MOH-000559)
South Africa	Ferzana Amod	University of KwaZulu-Natal	Nothing to declare
	Jonathan Carr	University of Stellenbosch	Nothing to declare
	Soraya Bardien	Stellenbosch University	National Research Foundation of South Africa[Grant Number 129249]
South Korea	Beomseok Jeon	Seoul National University Hospital	Nothing to declare
	Yun Joong Kim	Yongin Severance	Nothing to declare

		Hospital	
Spain	Esther Cubo	Hospital Universitario Burgos	Nothing to declare
	Ignacio Alvarez	University Hospital Mutua Terrassa	Nothing to declare
	Janet Hoenicka	Institut de Recerca Sant Joan de Deu	Fondo de Investigación Sanitaria, Instituto Salud Carlos III, Grant PI019/00126
	Katrin Beyer	Research Institute Germans Trias i Pujol	Nothing to declare
	Maria Teresa Periñan	Instituto de Biomedicina de Sevilla	Nothing to declare
	Pau Pastor	University Hospital Germans Trias i Pujol	Nothing to declare
Sudan	Sarah El-Sadig	Faculty of medicine university of Khartoum	Nothing to declare
Sweden	Kajsa Brolin	Lund University	Nothing to declare
Switzerland	Christiane Zweier	Inselspital Bern, University of Bern	Nothing to declare
	Gerd Tinkhauser	University Hospital Bern	Nothing to declare
	Paul Krack	Inselspital Bern, University of Bern	Nothing to declare
Taiwan	Chin-Hsien Lin	National Taiwan University Hospital	Nothing to declare
	Hsiu-Chuan Wu	Chang Gung Memorial Hospital	Nothing to declare
	Pin-Jui Kung	National Taiwan University	Global Parkinson's Genetics Program
	Ruey-Meei Wu	National Taiwan University Hospital	Minister of Science and Technology, Taiwan Government; National Taiwan University; Parkinson foundation (USA); Michael J. Fox Foundation
	Yihru Wu	Chang Gung Memorial Hospital	Nothing to declare
Tunisia	Rim Amouri	National Institute Mongi Ben Hamida of Neurology	Nothing to declare
	Samia Ben Sassi	Mongi Ben Hmida National Institute of Neurology	Nothing to declare
Turkey	A. Nazlı Başak	Koç University	Kirac Foundation and Koc Univ.
	Gencer Genc	Şişli Etfal Training and Research Hospital	Nothing to declare
	Özgür Öztop Çakmak	Koç University	Nothing to declare
	Sibel Ertan	Koç University	Nothing to declare
United Kingdom	Alastair Noyce	Queen Mary University of London	Prof. Noyce reports grants from Parkinson's UK, Barts Charity, Cure Parkinson’s, NIHR, Innovate UK, Virginia Keiley benefaction, Alchemab, Aligning Science Across Parkinson’s and Michael J Fox Foundation. Consultancy and personal fees from Astra Zeneca, AbbVie, Profile, Roche, Biogen, UCB, Bial, Charco Neurotech, uMedeor and Britannia.
	Alejandro Martínez-Carrasco	University College London	Global Parkinson's Genetics Program

	Anette Schrag	University College London	NIHR UCL/H Biomedical Research Centre
	Anthony Schapira	University College London	ASAP
	Camille Carroll	University of Plymouth	C Carroll receives salary from University of Plymouth, University Hospitals Plymouth NHS Trust and National Institute of Health Research; she has received advisory, consulting, and/or lecture fees from AbbVie, Bial, Lundbeck, Global Kinetics, Britannia and Medscape, and research funding from Parkinson's UK, Edmond J Safra Foundation, National Institute of Health Research and Cure Parkinson’s
	Claire Bale	Parkinson's UK	Nothing to declare
	Donald Grosset	University of Glasgow	Tracking Parkinson's (J-1101) is funded by Parkinson's UK
	Eleanor J. Stafford	University College London	Nothing to declare
	Henry Houlden	University College London	Nothing to declare
	Huw R Morris	University College London	Dr Morris is employed by UCL. In the last 12 months he reports paid consultancy from Roche and Amylyx ; lecture fees/honoraria - BMJ, Kyowa Kirin, Movement Disorders Society. Research Grants from Parkinson’s UK, Cure Parkinson’s Trust, PSP Association, CBD Solutions, Drake Foundation, Medical Research Council, Michael J Fox Foundation. Dr Morris is a co-applicant on a patent application related to C9ORF72 - Method for diagnosing a neurodegenerative disease (PCT/GB2012/052140)
	John Hardy	University College London	Nothing to declare
	Kin Ying Mok	Univeristy College London	Nothing to declare
	Mie Rizig	University College London	Nothing to declare
	Nicholas Wood	University College London	ASAP- CRN
	Nigel Williams	Cardiff University	Parkinson's UK
	Olaitan Okunoye	University College London	Nothing to declare
	Patrick Alfryn Lewis	Royal Veterinary College University of London	MJFF, UKRI (BBSRC, EPSRC), Parkinson’s UK, ASAP research network network
	Rauan Kaiyrzhanov	University College London	Nothing to declare
	Rimona Weil	University College London	Nothing to declare
	Seth Love	University of Bristol	Medical Research Council (UK) MR/T018569/1
	Simon Stott	Cure Parkinson's	Employee of Cure Parkinson's
	Simona Jasaityte	University College London	Nothing to declare
	Sumit Dey	Queen Mary University of London	Nothing to declare

	Vida Obese	University College London	Nothing to declare
USA	Alberto Espay	University of Cincinnati	Nothing to declare
	Alyssa O'Grady	The Michael J. Fox Foundation for Parkinson's Research	Nothing to declare
	Andrew B Singleton	National Institute on Aging	Michael J Fox Foundation for Parkinson's disease Research and Aligning Science Across Parkinson's Initiative
	Andrew K. Sobering	Augusta University / University of Georgia Medical Partnership	Nothing to declare
	Bernadette Siddiqi	The Michael J. Fox Foundation for Parkinson's Research	Nothing to declare
	Bradford Casey	The Michael J. Fox Foundation for Parkinson's Research	Nothing to declare
	Brian Fiske	The Michael J. Fox Foundation for Parkinson's Research	Nothing to declare
	Cabell Jonas	Mid-Atlantic Permanente Medical Group	Nothing to declare
	Carlos Cruchaga	Washington University	National Institutes of Health (R01AG044546 (CC), P01AG003991(CC, JCM), RF1AG053303 (CC), RF1AG058501 (CC), U01AG058922 (CC), RF1AG074007 (YJS)), the Chuck Zuckerberg Initiative (CZI), the Michael J. Fox Foundation (LI, CC), and the Department of Defense (LI-W81XWH2010849). The recruitment and clinical characterization of research participants at Washington University were supported by NIH P30AG066444 (JCM), P01AG03991(JCM), and P01AG026276(JCM).
	Caroline B. Pantazis	National Institutes of Health	Nothing to declare
	Charisse Comart	The Michael J. Fox Foundation for Parkinson's Research	Nothing to declare
	Claire Wegel	Indiana University	Nothing to declare
	Cornelis Blauwendraat	National Institutes of Health	Nothing to declare
	Dan Vitale	National Institutes of Health	Nothing to declare
	Deborah Hall	Rush University	Nothing to declare
	Dena Hernandez	National Institutes of Health	Nothing to declare
	Ejaz Shiamim	Kaiser Permanente	Nothing to declare
	Ekemini Riley	Coalition for Aligning Science	Nothing to declare
	Faraz Faghri	National Institutes of Health	F.F.'s participation in this research was supported in part by the Intramural Research Program of the NIH, National Institute on Aging (NIA), National Institutes of Health, Department of Health and Human Services; project number ZO1 AG000535, as well as the National Institute of Neurological Disorders and

			Stroke. F.F.'s participation in this project was part of a competitive contract awarded to Data Tecnica International LLC by the National Institutes of Health to support open science research.
	Geidy E. Serrano	Banner Sun Health Research Institute	Banner Sun Health Research Institute Brain and Body Donation Program of Sun City, Arizona for the provision of human biological materials. The Brain and Body Donation Program has been supported by the National Institute of Neurological Disorders and Stroke (U24 NS072026 National Brain and Tissue Resource for Parkinson's Disease and Related Disorders), the National Institute on Aging (P30 AG19610 and P30AG072980, Arizona Alzheimer's Disease Center), the Arizona Department of Health Services (contract 211002, Arizona Alzheimer's Research Center), the Arizona Biomedical Research Commission (contracts 4001, 0011, 05-901 and 1001 to the Arizona Parkinson's Disease Consortium) and the Michael J. Fox Foundation for Parkinson's Research
	Hampton Leonard	National Institute on Aging/National Institutes of Health	H.L.L is supported by a competitive contract awarded to Data Tecnica International LLC by the National Institutes of Health to support open science research
	Hirotaka Iwaki	Data Tecnica International	H.I is supported by a competitive contract awarded to Data Tecnica International LLC by the National Institutes of Health to support open science research
	Honglei Chen	Michigan State University	NIH/DoD/Parkinson Foundation/MSU Foundation/Gibby vs. Parky Foundation - No COI to disclose
	Ignacio F. Mata	Cleveland Clinic	Funding from MJFF and NIH
	Ignacio Juan Keller Sarmiento	Northwestern University	Nothing to declare
	Jared Williamson	Kaiser Permanente	Nothing to declare
	Jonggeol Jeff Kim	National Institutes of Health	Nothing to declare
	Joseph Jankovic	Baylor College of Medicine	Nothing to declare
	Joshua Shulman	Baylor College of Medicine / Texas Children's Hospital	Collection of samples and data at Baylor College of Medicine was supported by Huffington Foundation.
	Justin C. Solle	The Michael J. Fox Foundation for Parkinson's Research	Nothing to declare
	Kaileigh Murphy	The Michael J. Fox Foundation for Parkinson's Research	Nothing to declare
	Karen Nuytemans	University of Miami Miller School of Medicine	This work has been supported by the American Parkinson Disease Association and the Margaret Q. Landenberger Research Foundation.
	Karl Kieburtz	Beth Israel Deaconess Medical Center	Nothing to declare
	Katerina	North Shore University	Nothing to declare

	Markopoulou	Health System	
	Kenneth Marek	Institute for Neurodegenerative Disorders	Consultant for Michael J Fox Foundation, GE Healthcare, Roche, UCB, BIAL, Denali, Takeda, , Cerapsir, UCB, Biohaven, Neuron23, Aprinoia, Astellas, Calico, Genentech, Invicro
	Kristin S. Levine	Data Tecnica International	Nothing to declare
	Lana M. Chahine	University of Pittsburgh	Nothing to declare
	Laura Ibanez	Washington University	Nothing to declare
	Laurel Screven	National Institute on Aging	Nothing to declare
	Lauren Ruffrage	University of Alabama at Birmingham	Nothing to declare
	Lisa Shulman	University of Maryland	Nothing to declare
	Luca Marsili	University of Cincinnati	Nothing to declare
	Maggie Kuhl	The Michael J. Fox Foundation for Parkinson's Research	Nothing to declare
	Marissa Dean	University of Alabama at Birmingham	Dr. Dean is an investigator in studies funded by Abbvie, Inc., Hoffmann-La Roche, CHDI Foundation, Inc., Annexon, Inc., Retrophin, Inc, Neurocrine Biosciences, UniQure Biopharma B.V., Praxis Precision Medicines, Neuraly, Inc., Michael J. Fox Foundation for Parkinson's Research, and US Army Medical Research and Material Command (grant#W81XWH-18-1-0508). In addition, Dr. Dean receives support through the Huntington's Disease Society of American Centers of Excellence program.
	Mary B Makarious	National Institutes of Health	Nothing to declare
	Mathew Koretsky	National Institutes of Health	Nothing to declare
	Megan J. Puckelwartz	Northwestern University	Nothing to declare
	Miguel Inca-Martinez	Cleveland Clinic	Nothing to declare
	Mike A. Nalls	National Institutes of Health	M.A.N.'s participation in this project was part of a competitive contract awarded to Data Tecnica International LLC by the National Institutes of Health to support open science research. M.A.N. also currently serves as an advisor for Clover Therapeutics and Neuron23 Inc.
	Naomi Louie	The Michael J. Fox Foundation for Parkinson's Research	Nothing to declare
	Niccolò Emanuele Mencacci	Northwestern University	Nothing to declare
	Roger Albin	Universit of Michigan	P50NS123067
	Roy Alcalay	Columbia University	Dr. Alcalay is funded by the Micharl J. Fox Foundation and the Parkinson's Foundation. He received consultation fees from Avrobio, Caraway, GSK, Merck, Sanofi, Ono Therapeutics and Takeda
	Ruth Walker	James J. Peters	Research funding from the Department of Veterans

		Veterans Affairs Medical Center	Affairs (CSRD Merit Award CX002342), consulted for Teladoc, Inc. and received an honorarium from New York University (NYU) Medical Center.
	Sara Bandres-Ciga	National Institutes of Health	Nothing to declare
	Sohini Chowdhury	The Michael J. Fox Foundation for Parkinson's Research	Nothing to declare
	Sonya Dumanis	Aligning Science Across Parkinson's	Nothing to declare
	Steven Lubbe	Northwestern University	Nothing to declare
	Tao Xie	University of Chicago	Nothing to declare
	Tatiana Foroud	Indiana University School of Medicine	The Michael J. Fox Foundation for Parkinson's Research
	Thomas Beach	Sun Health Research Institution	Banner Sun Health Research Institute Brain and Body Donation Program of Sun City, Arizona for the provision of human biological materials. The Brain and Body Donation Program has been supported by the National Institute of Neurological Disorders and Stroke (U24NS072026 National Brain and Tissue Resource for Parkinson’s Disease and Related Disorders), the National Institute on Aging (P30 AG19610 and P30AG072980, Arizona Alzheimer’s Disease Center), the Arizona Department of Health Services (contract 211002, Arizona Alzheimer’s Research Center), the Arizona Biomedical Research Commission (contracts 4001, 0011, 05-901 and 1001 to the Arizona Parkinson's Disease Consortium) and the Michael J. Fox Foundation for Parkinson’s Research
	Todd Sherer	The Michael J Fox Foundation for Parkinson's Research	Nothing to declare
	Yeajin Song	National Institutes of Health	Nothing to declare
Vietnam	Duan Nguyen	Hue University	Nothing to declare
	Toan Nguyen	Hue University	Nothing to declare
Zambia	Masharip Atadzhanov	University of Zambia	Nothing to declare
